# Sulphated glycosaminoglycans and proteoglycans in the developing vertebral column of juvenile Atlantic salmon (*Salmo salar*)

**DOI:** 10.1007/s10695-015-0067-4

**Published:** 2015-05-12

**Authors:** Kirsten O. Hannesson, Elisabeth Ytteborg, Harald Takle, Grethe Enersen, Grete Bæverfjord, Mona E. Pedersen

**Affiliations:** Nofima AS, 1430 Ås, Norway; Nofima AS, 6600 Sunndalsøra, Norway

**Keywords:** Glycosaminoglycans, Proteoglycans, Vertebral development, Salmon

## Abstract

In the present study, the distribution of sulphated glycosaminoglycans (GAGs) in the developing vertebral column of Atlantic salmon (*Salmo salar*) at 700, 900, 1100 and 1400 d° was examined by light microscopy. The mineralization pattern was outlined by Alizarin red S and soft structures by Alcian blue. The temporal and spatial distribution patterns of different types of GAGs: chondroitin-4-sulphate/dermatan sulphate, chondroitin-6-sulphate, chondroitin-0-sulphate and keratan sulphate were addressed by immunohistochemistry using monoclonal antibodies against the different GAGs. The specific pattern obtained with the different antibodies suggests a unique role of the different GAG types in pattern formation and mineralization. In addition, the distribution of the different GAG types in normal and malformed vertebral columns from 15 g salmon was compared. A changed expression pattern of GAGs was found in the malformed vertebrae, indicating the involvement of these molecules during the pathogenesis. The molecular size of proteoglycans (PGs) in the vertebrae carrying GAGs was analysed with western blotting, and mRNA transcription of the PGs aggrecan, decorin, biglycan, fibromodulin and lumican by real-time qPCR. Our study reveals the importance of GAGs in development of vertebral column also in Atlantic salmon and indicates that a more comprehensive approach is necessary to completely understand the processes involved.

## Introduction

The vertebral column is the defining character of all vertebrates. It consists of an alternating pattern of vertebral bodies (centra), providing strength and support, and intervertebral regions (IVR), providing flexibility and resistance to compression. The vertebral column develops from notochord, a flexible rod-like structure derived from the mesoderm. In contrast to the notochord in mammals, where only remnants exist in the intervertebral disc in adulthood, the notochord in many teleosts, including Atlantic salmon (*Salmo salar*), persists in the entire length of the vertebral column throughout the whole lifespan. During the embryonic and larval stages, the notochord is the main axial support playing essential roles in the development of the vertebral column by serving structural as well as signalling roles for patterning, also of surrounding tissue. Throughout the early yolk-sac stage, the notochord of Atlantic salmon is unsegmented with a uniform notochord sheath of even thickness surrounded by the sclerotome (Grotmol et al. 2003, 2005). After hatch, when the salmon larvae start to move, a stronger backbone is needed and mineralization of the notochord and sclerotome starts (Nordvik et al. 2005). Mineralization begins already at 650 d° in Atlantic salmon, when matrix in the outer half of the ventral notochordal sheath becomes mineralized (the chordacentrum). During the growth period from 800 to 1400 d°, the main events have occurred, resulting in the architecture of the adult salmon vertebral column (Nordvik et al. 2005).

Many studies have focused on early development of the vertebral column. In different teleost species, a pool of mesenchymal stem cells (MSCs) and neural and haemal arch cartilages surround the external elastic membrane of the notochord at 300 d° are described (Koumans et al. 1990; Koumoundouros et al. 1999; Potthoff et al. 1986; Powell and Tucker 1992). The process where these cells differentiate and develop into specific tissue types and body parts is complicated, highly controlled and orchestrated by a number of signals, including external (e.g. temperature and nutrition) and internal (e.g. hormones and transcription factors). The sequential order of these signals is of great importance for correct and normal development. The proteoglycans (PGs) represent a ubiquitous family consisting of more than 30 members that are involved in many biological processes, ranging from structural and mechanical foundation characteristics to biochemical involvement in mineralization, growth, differentiation and metabolism. PGs are together with collagen the template for mineralisation, and their structure and temporal presence are of great importance for correct and normal development. In mammals, proteoglycans (PGs), expressed by notochord-like cells in the intervertebral disc, are reported to influence disc integrity and function (Aguiar et al. 1999; Erwin and Inman 2006; Iwasaki et al. 1999). In both mammals and birds, the composition of PGs and the structure of the glycosaminoglycans (GAGs) attached are known to play pivotal roles for skeletal development (Aszodi et al. 2000). PGs in matrix are divided into two major groups, the hyalectans and the small leucine-rich proteoglycans (SLRPs). The former is able to aggregate with hyaluronic acid and link proteins together, forming huge hydrated space filling polymers. The SLRPs, on the other hand, are smaller extracellular molecules that bind to growth factors, collagens and other matrix molecules.

The common feature for the PG members is a protein core with one or more covalently attached sulphated carbohydrate side chains (glycosaminoglycans, GAGs). The core protein of the PGs ranges in molecular sizes from around 10 kDa to above 500 kDa, and each protein is encoded by a lonely gene belonging to different gene families (Iozzo 1998). The multifunctionality is dependent on tissue, cell type and/or metabolic context, and where the carbohydrate chains of the proteoglycans with different structurally modifications fine tunes the proteoglycans interactions with a variety of ECM components and cellular ligands, such as chemokines, growth factors, adhesion molecules and collagens (Hardingham and Fosang 1992). Structurally, the GAGs are composed of repeating disaccharide units consisting of a hexosamine and an uronic acid residue, forming long linear chains. Depending on the type of the disaccharide building blocks, the GAGs are divided into three major groups: the chondroitin/dermatan sulphate, (CS/DS), the keratan sulphate (KS) and the heparan sulphate (HS). The first two groups, CS/DS and KS represent major GAGs of the extracellular matrix (ECM) in mammals and birds. The disaccharide of CS consists of d-glucuronic acid (GlcA) and *N*-acetyl galactosamine (GalNAc). The GalNAc may be non-sulphated (C-0-S) or sulphated in the 4th (C-4-S) or 6th (C-6-S) position. In DS, the GluA is epimerized to iduronic acid (IdoA). KS is composed of galactose and GluNAc and is the only GAG that does not contain hexuronic acid. Variations in the amount and position of the sulphate groups in the GAG chains and amount of epimerization of glucuronic acid residues influence the type and strength of interactions with other extracellular components.

In the present study, we describe the distribution of sulphated GAGs in the vertebral column of Atlantic salmon during early development by light microscopy methods. Stages in the development (700, 900, 1100 and 1400 d°) were selected to follow the process from notochord to mineralized vertebral column. The different GAG subtypes and sulphate structures were analysed by immunohistochemistry using monoclonal antibodies against, C-0-S, C-4-S, C-6-S and DS after enzymatic treatment (Caterson et al. 1985; Couchman et al. 1984). Monoclonal antibodies against highly sulphated KS were also included. We also compared the distribution of the GAG types in salmon with normal and pathologic vertebral development collected at a later stage of development and approached the number and size of the macromolecules carrying the GAG epitopes by western blotting. We have previously reported the transcription of proteoglycans in salmon with malformations (Pedersen et al. 2013). In the present study, the transcription of PGs known to carry the C-0-S, C-4-S, C-6-S, DS and KS epitopes was analysed in salmon with healthy development with real-time qPCR. A changing composition of GAGs during spinal segmentation, when tissues are organizing and differentiating, is relevant for the understanding of vertebral development in fish, both under normal conditions and during pathogenesis. Our results reveal new and important distribution patterns of macromolecule components during vertebral development in fish.

## Materials and methods

### Sampling

Fish originating from the SalmoBreed strain was collected at the Nofima research station at Sunndalsøra, Norway, in 2007. Eyed salmon eggs were incubated at 8 °C until hatching at approximately 500 d°. Yolk-sac fry were maintained at 8 °C until time of first feeding at 850 d°. At time of first feeding, fry were transferred to circular fibre glass tanks (∅ 0.5 m) supplied with water at a controlled temperature of 12 °C and given commercial feed in excess. Fish tanks were inspected daily. At sampling, fish were euthanized (tricaine methane sulphonate, Pharmaq, Overhalla, Norway) before dissection. Notochord and surrounding tissue were dissected from salmon at necropsy, at the following developmental stages: 700, 900 (0.2 g), 1100 (0.5–0.6 g) and 1400 d° (1.3–1.4 g), frozen in liquid nitrogen and kept at −80 °C until use. Developmental stages were classified as day degrees (d°), which is defined as the sum of daily ambient water temperatures in degrees for each day of development. Radiography of 2 and 15 g fish was performed at Nofima radiology laboratory at Sunndalsøra, Norway. The setup was semi-digital, with a mammography X-ray source (MS Giotto, Bologna, Italy) and with the use of reusable image plates with mammography resolution, and exposure at 22 kV/40 mAs. At 2 and 15 g size, 40 and 100 fish per tank, respectively, were X-rayed. Images were read and transferred to the computer in a plate reader (FCR Profect, Fuji Medical Inc., Japan). The images were automatically processed before storage, adjustment of brightness and contrast, equalization of exposure and highlighting of edges (Fuji CR Console software, Fuji Medical Inc., Japan). Based on evaluation of the digital images, fish with no vertebral malformations and fish with severe vertebral malformations were identified and subsequently euthanized and selected for analysis; for histological analyses, samples were stored 24 h in PFA before dehydrated in a graded series of ethanol; for RNA and western blot analysis, samples were snap frozen in liquid nitrogen and stored at −80 °C before further analysis.

### Sectioning and mounting

Serial sections of 5 μm of OCT-embedded tissue were cut in the para-sagittal and transversal plane of the notochord and vertebral columns of fish (*n* = 3) collected at 500, 900, 1100 and 1400 d° of development and of 15 g weight (*n* = 4) with and without deformities, in a cryostat (Leitz 1720 Digital, Leica Instruments GMBH, Heidelberg, Germany), mounted on poly-l-lysine coated glass slides and kept at −20 °C. All sections were analysed microscopically, using a LEICA DMLB microscope (Leica Microsystems Nussloch GmbH, Germany) and photographed in a spot RT Color Camera (Diagnostic Instruments Inc. Burroughs Sterling Heights, Michigan Heights). Demineralization was not performed in order to avoid destruction of the antigenic epitopes.

### Histological staining

Sections were stained with haematoxylin (Riedel-de-Haen, Germany) combined with erythrosine B (Aldrich-Chemie, Germany) and saffron to outline general architecture of the vertebrae. In brief, sections were stained in haematoxylin for 5–10 min, washed in water for 20 min, and fixed in 0.25 % hexamine for 3 min before stained in erythrosine B for 4 min. Finally, the sections were dehydrated in ascending concentrations of alcohol before staining with saffron for 3 min. To visualize mineralized regions, sections were stained with Alizarin red S (pH 4.2) for 5 min. To examine the distribution of sulphated GAGs, a solution containing 0.05 % Alcian blue 8GX (Gurr Biological Stains, BDH, Poole, UK) in 0.2 M Na-acetate buffer pH 5.8 added 0.4 M MgCl_2_, was used. At a concentration of 0.4 M MgCl_2_, only negatively charged groups such as sulphated GAGs stain (Scott and Dorling 1965; Scott and Dorling 1965). The sections were immersed in the staining solution at room temperature with gentle shaking overnight, rinsed in running water, dehydrated in absolute ethanol, cleared in xylene, and mounted in Eukitt. All stained sections were microscopically examined with a Leica DMLB microscope (Leica Microsystems Nussloch GmbH, Germany).

### Immunohistochemistry

Immunohistochemical identification of the different GAGs CS/DS and KS chains was obtained with the following mAbs: mAb 1B5 for detection of C-0-S, mAb 2B6 for detection of C-4-S and mAb 3B3 for detection of C-6-S (Seikagaku America, MA). The identification of the different GAG types was performed on the same individual and repeated on different fish (*n* = 3). To generate the antigenic epitopes for detection of CS/DSPGs, the tissue samples were digested with chondroitinase ABC lyase (cABC) from *Proteus vulgaris* (0.5 units/mL) (EC 4.2.2.4, Sigma-Aldrich Chemie Gmbh, Steinheim, Germany) in 0.1 M Tris–HCl buffer pH 8 for 2 h at 37 °C (Caterson et al. 1982; Yamagata et al. 1968). To detect only CS, sections were treated with chondroitinase AC II, pH 6.0 for 2 h at 37 °C. Digestion with cABC was also performed on the samples to be examined for KS by immunohistochemistry to increase the penetration of the KS antibodies. The enzyme is inactive with the KS chains (Hamai et al. 1997). After cABC treatment, non-specific binding sites were blocked using 5 % teleost gelatin (Sigma-Aldrich) in PBS-added normal serum from horse (Vectastain Universal Elite ABC kit, Vector Laboratories Inc., Burlingame, USA). The sections were then incubated overnight at 4 °C with the following monoclonal antibody concentrations: 3B3 (1:200), 1B5 (1:400), 2B6 (1:400) and 5D4 (1:200), for detection of C-6-S, C-0-S, 4 sulphated CS/DS and KS, respectively. The mAbs were diluted in PBS-added 5 % teleost gelatin or BSA and 0.005 % Tween-20 (Sigma-Aldrich). Immunostaining was performed using an immunoperoxidase system (Vectastain Universal Elita ABC kit, Vector Laboratories) according to the manufacturer’s recommendations. Sections were counterstained with haematoxylin. Non-immune serum from the same species as the primary antibody was used in control experiments. Non-specific binding of the secondary antibody was tested by replacing the latter with dilution buffer. Control tests were also performed on sections without digestion with chondroitinase ABC. A Spot RT Color Camera (Diagnostic Instruments Inc. Burroughs Sterling Heights, Michigan Heights) photographed the sections with a Leica DMLB microscope.

### RNA isolation, cDNA synthesis and real-time qPCR

Vertebrae dissected from 12 fish of 2 g (*n* = 12) and 15 g fish (*n* = 12) size were homogenized in liquid nitrogen before total RNA was isolated using Trizol^®^ (Invitrogen, MD, USA) and reagents from the RNAeasy Micro to Midi Kit^®^ (Qiagen, Hilden, Germany). All samples were DNase I treated (Invitrogen), and RNA quality was assessed by 1 % agarose gel. One microgram of RNA was subjected to reverse transcriptase (RT) reaction by TaqMan Gold RT-PCR kit (Applied Biosystem, CA, USA). The samples were diluted 5× before application of samples (in triplicates) to real-time PCR analysis in an ABI prism 7900HT sequence detection system (Applied Biosystem), using TaqMan 100 rx PCR core master kit (Applied Biosystem). At first, uracil-N-glycosylase (UNG) treatment at 50 °C for 2 min and UNG inactivation at 95 °C for 10 min were performed, followed by amplification of cDNA by 40 two-step cycles (15 s at 95 °C for denaturation of DNA, 1 min at 60 °C for primer annealing and extension). Cycle threshold (*C*_t_) values were obtained graphically (Applied Biosystem, Sequence Detection System, Software version 2.2). Gene expression was normalized to elongation factor 1 alpha (EF-1α), and Δ*C*_t_ values were calculated. Comparison of gene expression between samples (15 and 2 g) was derived from subtraction of Δ*C*_t_ values between the two samples to give a ΔΔ*C*_t_ value, and relative gene expression calculated as $2^{{ - {\Delta \Delta }C_{\text{t}} }}$ (fold difference). The primers and TaqMan probes (Table 1) (5′ labelled-6-FAM and 3′ quencher TAMRA) for real-time PCR amplification of decorin (GenBank accession no. NM001173562.1), biglycan (GenBank accession no. FJ799991.1), lumican (GenBank accession no. NM001140062.1), aggrecan (GenBank accession no. FJ 179677), fibromodulin (GenBank accession no. FJ195618) and internal standard EF-α (Genbank accession no. AF321836) were designed by using Primer Express Program (Applied Biosystem).

**Table 1:** Primer and probe sequences

Gene	Nucleotide sequence
Decorin	Fw-5′-CATTCCCCTGGGCAAGCT-3′
	Rw-5′-TTGGCAGGCATTTCCTTGAG-3′
	P-5′-CAGCGCCTCTACCTCTCCAAGAACC-3′
Biglycan	Fw-5′-CAGGTGGTCTACCTCCATTCCA-3′
	Rw-5′-AAAGCCTCTTGGGCAGAAGTC-3′
	P-5′-CAGCATCAGCAAGGTGGGAGTGGA-3′
Lumican	Fw-5′-AAGTTCTCTGGCCCTCTGAACTAC-3′
	Rw-5′-AGGTCGGCATGTGTGAGGTT-3′
	P-5′-ACTGAAGCACCTGCGTCTGG-3′
EF-α	Fw-5′-GCCAGATCTCCCAGGGCTAT-3′
	Rw-5′-TGAACTTGCAGGCGATGTGA-3′
	P-5′-CCCCTGTGCTGGATTGCCATAC-3′
Fibromodulin	Fw-5′-TGACCATCCTGTATCTCCATGACA-3′
	Rw-5′-TGAGTGAGTTCAACCCCCTAAGA-3′
	P-5′-CGCTTTGACGGAGATGGGCACA-3′
Aggrecan	Fw-5′-TGGCGGCCGAACCA-3′
	Rw-CCGTTCTCGTGCCAGATCAT-3′
	P-5′-CCGGATAACTACTTTAACTCTGGAGAAGACTGTGTGG-3′

### Protein extraction and western blot

Proteins from 15 g vertebrae of five fish (*n* = 5) were isolated from the residual fractions after the RNA isolation using Trizol^®^ (Invitrogen) according to the manufacturer’s protocol. Protein concentrations were measured with a commercial kit at 750 nm (*RC DC* Protein Assay, Bio-Rad, USA) in a spectrophotometer (Ultrospec 3000, Pharmacia Biotech) using BSA as a standard. The samples were treated with 0.5 units chondroitinase ABC (*P. vulgaris*, EC 4.2.2.4, Sigma) in 0.05 M Tris–HCl pH 7.5 for generation of epitopes of C-4-S, DS, C-6-S, C-0-S and KS over night at 37 °C. Equal amounts of chondroitinase ABC-treated samples (20 μg) were subjected to SDS-gel electrophoresis using 4–12 % Bis–Tris gels (Invitrogen), SDS-PAGE NuPAGE^®^ MOPS SDS running buffer (Invitrogen) and Novex Xcell II apparatus (Invitrogen). Transfer of the proteins to nitrocellulose membrane was performed by western blotting in transfer buffer consisting of 10 % methanol with 10 mM 3-cyclohexylamino-1-propanesulphonic acid (ACPS) pH 11 for 1 h at 80 V. Non-specific binding sites were blocked using 5 % teleost gelatin (Sigma) in 0,1 M Tris-saline, pH 7,4. The nitrocellulose membrane were then probed with monoclonal antibodies against C-4-S, C-6-S, C-0-S and KS (the same antibodies as used in the immunohistochemistry) diluted 1:1000 in TBS-added 0.5 % teleost gelatin. Alkaline phosphatise (AP)-conjugated secondary antibodies [anti-mouse IgG(H + L), Promega, WI, USA] diluted 1:7500 were then applied on the membranes. Immunoreactive bands were revealed with Novex^®^ AP (BCIP/NBT) colour development substrate (Invitrogen), and reaction stopped by distilled water.

### Statistical analysis

Statistical analysis was performed using a two-tailed, unpaired Student’s *t* test. *P* values <0.05 were considered statistically significant.

## Results

### Histological examination

**Fig. 1:**
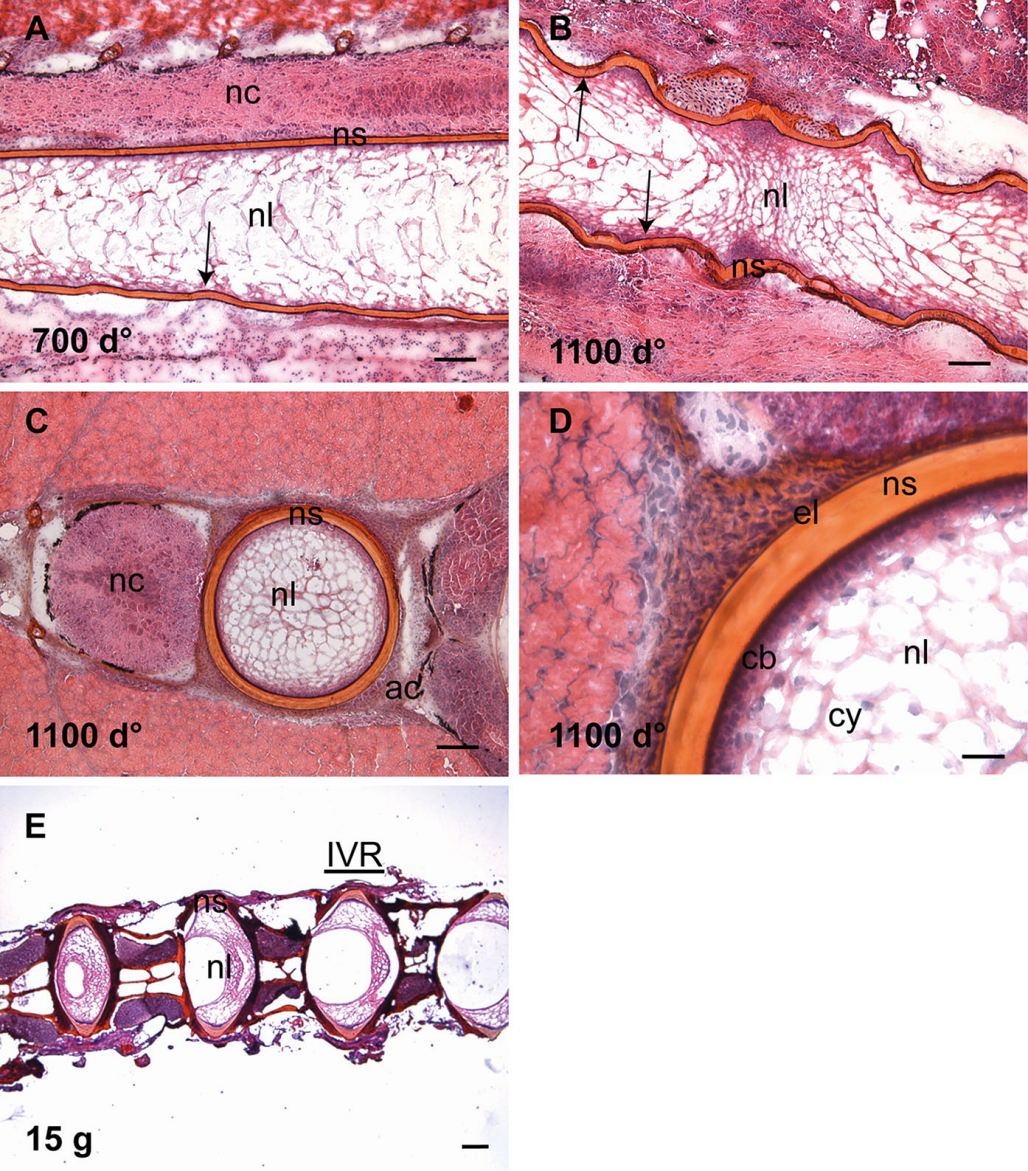
HE-added saffron staining of spinal columns. **a** At 700 d°, bulging of the ventral part of the notochord begins to emerge (*arrow*). **b** At 1100 d°, extensive bulging of the notochord is seen at both ventral and dorsal side and the metameric pattern of the future vertebral column becomes evident. **c** Transverse section at 1100 d° and **d** a higher magnification of **c**, illustrating the lumen of the notochord, the chordoblast and the chordocytes. **e** The adult spine in 15 g fish with vertebral bodies separated by intervertebral regions. *Scale bar* 100 µm; *nl* notochord lumen, *nc* neural cord, *ac* arch centra, *cb* chordoblast, *cy* chordocytes, *ns* notochordal sheath, *el* elastic lamina

The architecture of the notochord and the vertebral column in the time span studied was outlined by HE staining-added saffron, here illustrated with longitudinal and transverse sections of salmons obtained at 700 and 1100 d° (Fig. 1a–d). At 700 d°, some bulging of the ventral part of the notochord begins to emerge (arrow, Fig. 1a), and at 1100 d°, extensive bulging of the ventral as well as dorsal side of the notochord is seen (arrows, Fig. 1b), and the metameric pattern of the future vertebral column becomes evident. The transverse section of the fish at 1100 d° (Fig. 1c) outlines the neural chord (nc), the notochord sheath (ns), notochord lumen (nl), and the cartilaginous arches (ac). Figure 1d shows the transverse section at a higher magnification and the characteristic cartilaginous tissue of the arches separated from the sheath by the external elastic lamina (el). In the lumen of the notochord, the chordoblast (cb) layer and the chordocytes (cy) are clearly outlined. The adult spine, showing the fully developed vertebral bodies and the intervertebral regions (IVRs), is shown in 15 g fish (Fig. 1e).

### Mineralization of the developing vertebrae

**Fig. 2:**
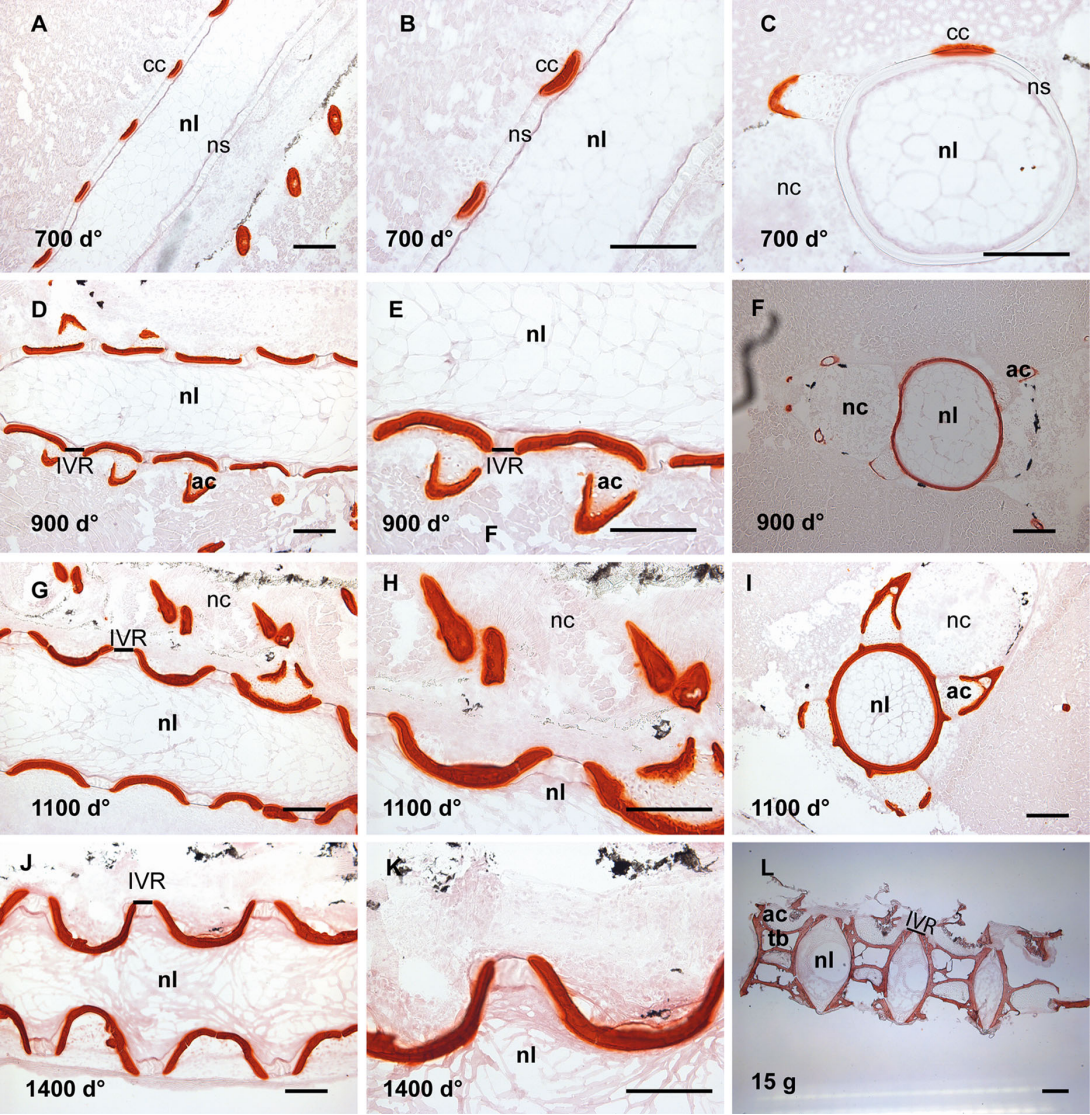
Alizarin red S staining of spinal column. **a** Longitudinal section at 700 d°, showing staining of the chordacentra in a repetitive pattern on one side of the notochord. **b** Higher magnification of **a** where the chordacentra show graded staining, weaker towards the notochord lumen. **c** Transverse section showing mineralization of the chordacentra on the lateral side of the notochord and the neural arcualia. **d** Longitudinal section at 900 d° where mineralization of the chordacentra proceeds. This is enlarged in **e**, showing the curved shape of the chordacentra. **f** Transverse section showing mineralized tissue surrounding the entire notochord. **g**, **h** Longitudinal sections at 1100 d° showing further development of the vertebral column with emerging vertebral endplates and unmineralized IVR. The concave shape of the vertebral bodies is visible. **i** Transverse section showing a thick mineralized ring with the perichordal bony arcualia. **j** Longitudinal section at 1400 d°, mineralization of the notochord is completed separated by unmineralized IVRs, enlarged in **k**. **l** Staining of the vertebral column from fish at 15 g size, with vertebral bodies containing amphicoel and the trabecular bone, separated by the IVRs. *Scale bar* 100 µm; *nl* notochord lumen, *nc* neural cord, *ac* arch centra, *ns* notochordal sheath, *tb* trabeculae bone, *IVR* intervertebral regions

Alizarin red S staining was used to trace the mineralization process as illustrated in Fig. 2. At 700 d°, the initial mineralization of the chordacentra was revealed in a repetitive way beneath the external elastic lamina in one side of the notochord (Fig. 2a, b), visualized in Fig. 2c on the lateral side of the notochord. The peripheral part of the chordacentra showed a deeper red colour, fainting towards the notochord lumen (Fig. 2b). The dorsal side of the sheath did not show any mineralization, neither in the longitudinal nor in the transverse sections. But mineralization of the upper, dorsal rim of the neural arcualia had started (Fig. 2c). At 900 d°, the mineralization of the chordacentra proceeded in both caudal and cranial directions in the ventral and now also in the dorsal notochordal sheath (Fig. 2d, e), surrounding the entire notochord (Fig. 2f). The mineralization of the neural and haemal arches also emerged (Fig. 2f). At 1100 d°, the mineralization of the sheath in the areas of chordacentra was completed in the full thickness of the sheath and the concave shape of the future vertebral bodies appeared (Fig. 2g, h). Hence, the template for the unmineralized intervertebral region (IVR) was established (Fig. 2g, h). The transverse section (Fig. 2i) shows a thicker red-coloured ring with perichordal bony projections, indicative of fully mineralized sheath in the transversal plane. In addition, the mineralization zones both in the periphery and the central areas of the arcualia were broader (Fig. 2i). At 1400 d°, the metameric segmentation of the notochord was completed, showing the contour of the spine as present in the fully developed salmon with vertebral bodies separated by unmineralized IVRs (Fig. 2j, k), representing the growth zones for vertebral length and thickness. Staining of the vertebral column from a 15 g fish with Alizarin red S showed that the segmentation was completed, with the bone of the vertebral amphicoel and the trabeculae, separated by the IVR (Fig. 2l).

### Distribution of sulphated GAGs in the developing vertebrae

**Fig. 3:**
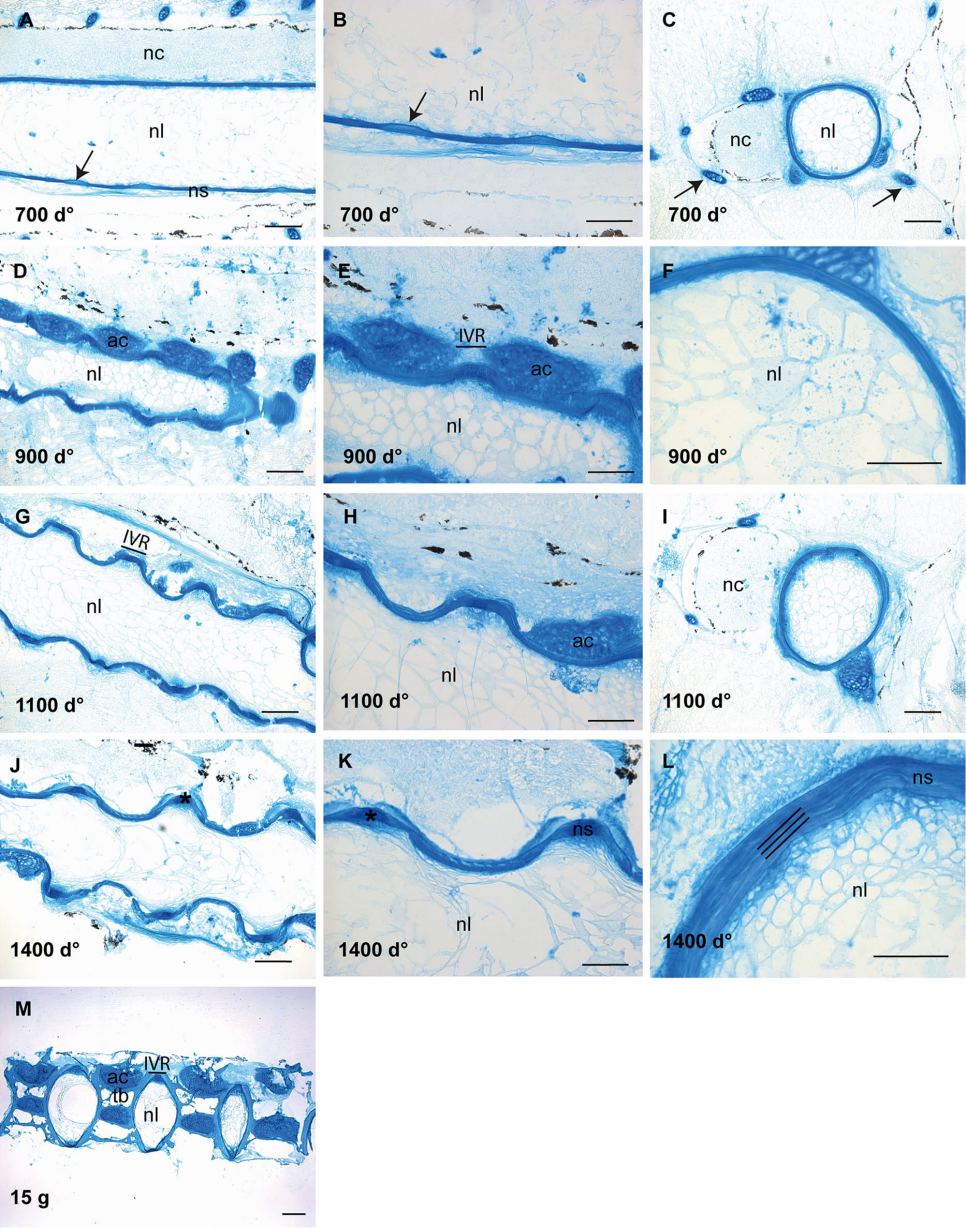
Alcian blue staining of the vertebral column. **a** Longitudinal section at 700 d°, showing a strong staining of notochordal sheath and the arcualia. Higher magnification in (**b**), where the metameric staining pattern of the notochord sheath is evident (*arrow*). **c** Transverse section showing a faint-stained network in the lumen, indicating the presence of GAGs in chordoblasts and chordocytes at 700 d°, the templates for development of the neural and haemal arches showed strong staining (*arrows*). **d**, **e** Longitudinal sections at 900 d° showing further growth and development of the future IVRs and the cartilaginous arches. **f** Transverse section showing a continuous staining of the notochord sheath. **g** Longitudinal section at 1100 d° where the notochord curls along with the formation of the chordacentra, enlarged in **h** and the transverse section in **i** showing a layered staining pattern of the notochord sheath. **j** Longitudinal section at 1400 d°, the IVRs of the notochordal sheath stained dark, enlarged in **k** where the templates for the notochord endplates emerge. In the transverse section, the layered structures were clearly outlined in the notochordal sheath (triple lines in **l**). **m** Longitudinal section of 15 g salmon showing a full-maturated vertebral column with strong staining for GAGs in mineralized and unmineralized compartments. *Scale bar* 100 µm; *nl* notochord lumen, *nc* neural cord, *ac* arch centra, *ns* notochordal sheath, *tb* trabeculae bone, *IVR* intervertebral regions

Alcian blue staining of the consecutive sections showed the distribution of sulphated GAGs in the notochord and surrounding tissue (Fig. 3). Sections collected from fish at 700 d° showed a strong and uniform blue staining of the notochordal sheath, arcualia and a weaker staining of the surrounding tissue (Fig. 3a–c). In the ventral part of the sheath where mineralization of the chordacentra was observed, regularly arranged areas of weaker staining appeared, demarcated on both sides by darker blue colour (arrow, Fig. 3a, b). In the transverse sections of notochord (Fig. 3c), a blue somewhat faint-stained network was evident in the lumen, showing the presence of GAGs also in chordoblasts and chordocytes. Furthermore, the templates for both neural and haemal arches were clearly outlined at 700 d° (arrows, Fig. 3c). In sections collected from fish at 900 d°, a change in the staining pattern appeared: a darker blue staining was seen in the areas for development of the future IVRs (Fig. 3e). The staining intensity of the chordocyte network of the notochord lumen increased. Although the cartilaginous appearance of the arcualia (ac) became visible at 700 d° (Fig. 3c), the cartilaginous nature of the growing arcualia was more evident at 900 d° (Fig. 3d–f). No major differences were observed in the distribution pattern of GAGs in sections collected at 1100 d° (Fig. 3g–i). At 1400 d°, the IVRs of the sheath with the chordoblasts and chordocytes exhibited a darker blue colour (Fig. 3j–l). In the IVRs, endplates were surrounded by a darker blue layer (Fig. 3k). In the transverse section at 1400 d°, concentric lamellar structures appeared in the sheath (Fig. 3l). The results after staining longitudinal sections from 15 g salmon with Alcian blue showed strong staining in the various compartments of the vertebral column such as the cartilaginous tissue of the amphicoel, the IVRs with the notochordal sheath and the bony endplates and the chordoblast layer as well as the chordocytes in the lumen (Fig. 3m), revealing an abundance of sulphated GAGs in mineralized as well as unmineralized tissue of the salmon vertebral column in adulthood.

### Distribution of GAGs in the developing vertebral column

To trace the contribution of various GAG subtypes to the staining patterns, C-4-S, DS, C-6-S, C-0-S and KS were immunolocalized. Below is the specific pattern obtained by each subtype.

### Chondroitin 4-sulphate (C-4-S) and dermatan sulphate (DS)

**Fig. 4:**
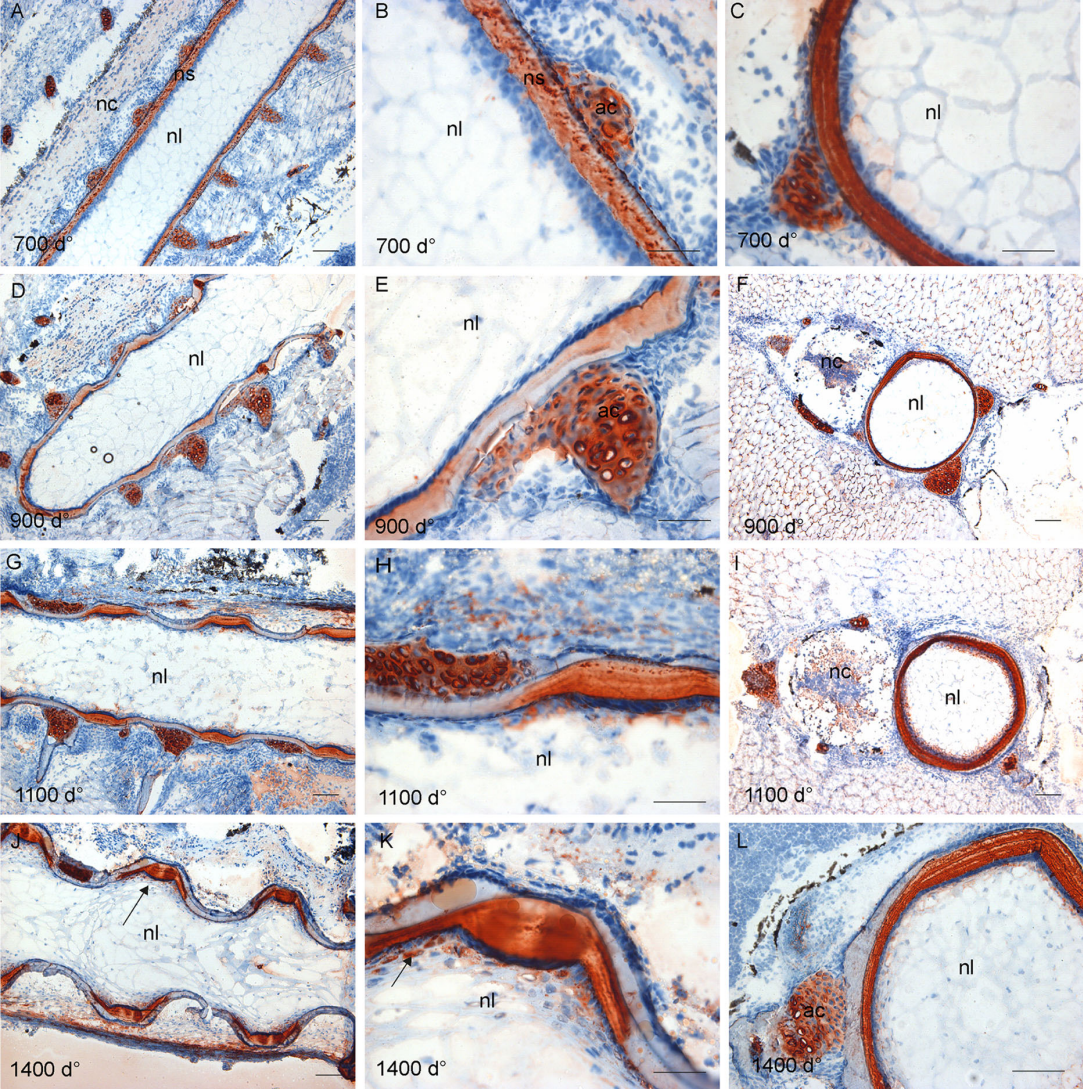
Distribution of chondroitin 4-sulphate (C-4-S) and dermatan sulphate (DS). **a** Longitudinal section at 700 d° showing strong staining of the notochordal sheath and the perichordal cartilage. The staining of notochordal sheath appeared granular, as clearly outlined in **b**. **c** The transverse section showing a more continuous staining of the sheath and staining of the cartilaginous arches. **d** Longitudinal section at 900 d° showing the staining of the notochordal sheath become weaker in areas of developing chordacentra, enlarged in **e**. **f** Transverse section showing stronger staining towards the lumen. **g** Longitudinal section at 1100 d°, the strong staining persisted in developing IVRs, higher magnification in **h**, showing a granular staining pattern in the external region of the IVR in contrast to the layered appearance in the central part. **i** Transverse section showing stronger staining towards the lumen. **j**, **k** Longitudinal sections at 1400 d°, showing a strong staining of the IVR in the central region and distinct, labelled wings extending towards the mineralizing zones (*arrow*), enlarged in **k**. Some staining was also seen in the chordoblast layer (*arrow*). **l** Transverse sections showing strong, lamellar staining of the notochordal sheath. *Scale bar* 100 µm; *nl* notochord lumen, *nc* neural cord, *ac* arch centra, *ns* notochordal sheath

The mAb 2B6 binds to both C-4-S and DS when the sections are treated with chondroitinase ABC but only to C-4-S when the sections are treated with chondroitinase ACII, as described in materials and methods. Subsequently, C-4-S was identified by direct observations of stained structures after treatment with the latter, whereas DS containing structures were identified as those that were stained with the former and not the latter. A different expression pattern of C-4-S and DS epitopes during development of the salmon spine was detected (Fig. 4). Using mAb 2B6 + cABC lyase for detection of both C-4-S and DS containing structures, strong staining of both the notochordal sheath and the perichordal cartilage was found in sections at 700 d° (Fig. 4a–c). In the longitudinal section (Fig. 4b), staining of the notochordal sheath appeared granular, whereas in the transverse section (Fig. 4c), heavily stained notochordal sheath showed a more uniform appearance, with traces of concentric lamellae. At 900 d°, the staining of the sheath became weak in the mineralized areas and the granular staining pattern became more uniform (Fig. 4d, e). A strong label persisted in developing IVRs that appeared at 1100 d° in parallel layers (Fig. 4g, h). However, in the outer rims of these regions, more granular staining could be seen (Fig. 4h), thus indicating a different organization pattern of C-4-S/DS (Fig. 4h). A faint staining of the mineralized part of the sheath was also evident and the chondrogenic arches stained strongly at all developmental stages. At 1400 d°, a well-organized staining pattern appeared in the IVR, showing a strong and uniform staining of the central region with distinct, labelled wings extending towards the mineralizing zones (arrow, Fig. 4j) was visualized. Furthermore, staining was seen in the chordoblast area (arrow, Fig. 4k). Strong staining was seen in the sheath and in the arches in the transverse sections (Fig. 4l).

**Fig. 5:**
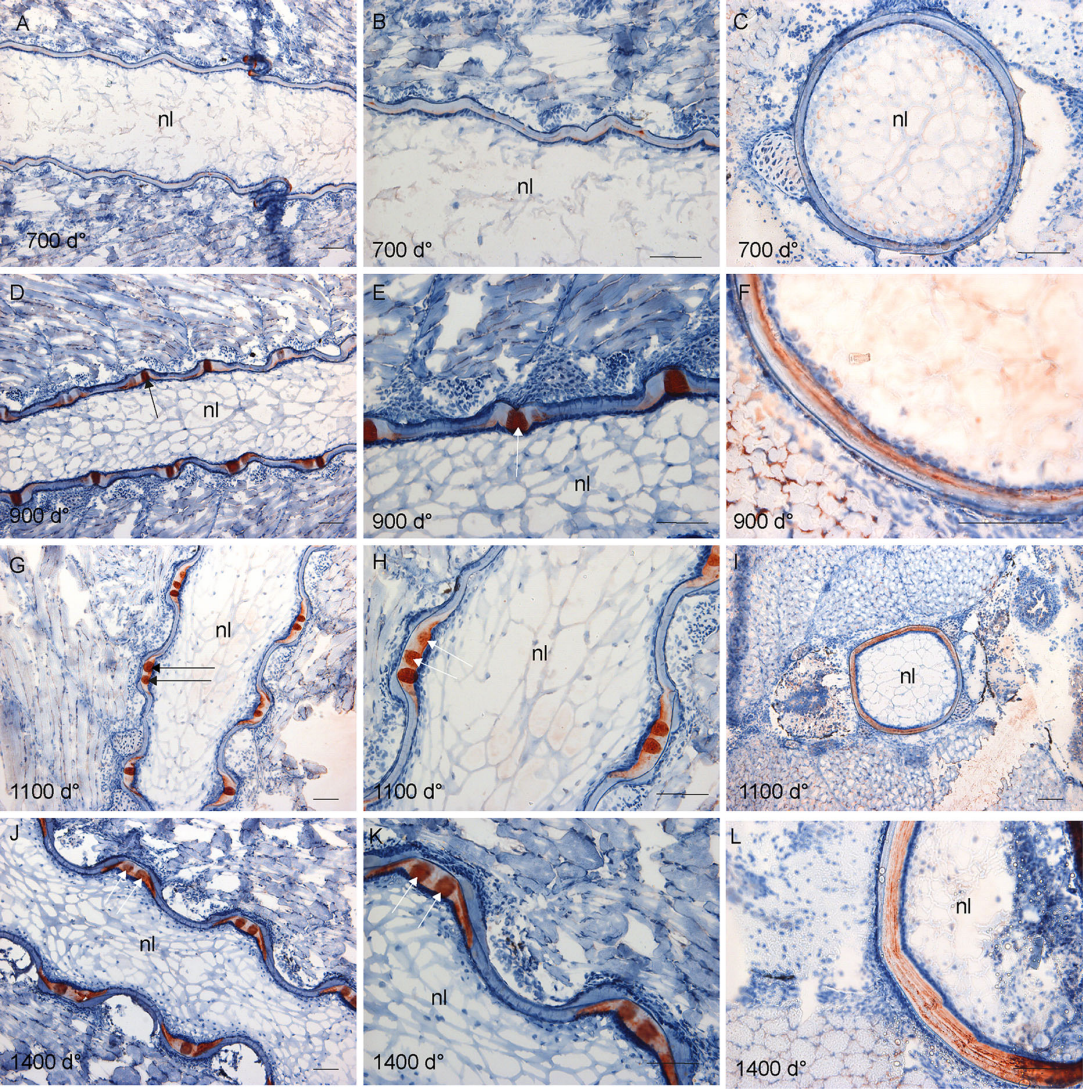
Distribution of chondroitin 4-sulphate (C-4-S) in the salmon spine. In the longitudinal sections **a**, **b**, and the transverse section, **c** collected at 700 d°, staining could hardly be seen. **d**, **e** Longitudinal sections at 900 d°, staining in developing IVRs appeared, very strongly in the central part (*arrows*), enlarged in **e**. **f** Transverse section showing that staining appeared in graded levels towards the lumen. **g** At 1100 d°, staining showed two–three distinct globes in the IVRs, enlarged in **h** and indicated by *arrows*. In addition, staining was seen in the layer of IVR towards lumen. **i** Transverse section showing that C-4-S was present in the notochord. **j** Longitudinal section at 1400 d°, the pattern of distinct globes and wings became more evident (*arrows*), enlarged in **k**. **l** Transverse section showed the distinct lamellar pattern of the notochordal sheath. No staining was seen in the lumen of the notochord at any stages of development. *Scale bar* 100 µm; *nl* notochord lumen

Replacing the enzyme cABC lyase with cACII produced a different labelling pattern (Fig. 5). With this enzyme, only C-4-S creates the immunostaining. At 700 d°, staining could hardly be seen in the notochordal sheath and no staining was found in chondrogenic tissue (Fig. 5a–c). This indicates that the dominating GAG in the notochordal sheath at 700 d° was DS. However, at 900 d°, an increase in the expression of C-4-S was detected, mainly restricted to the developing IVRs of the notochordal sheath, appearing as dark brown circular structures placed centrally in the IVR (Fig. 5d–f). At 1100 d°, the staining clearly showed two distinct spots in the IVRs (arrows, Fig. 5g, h), which persisted at 1400 d° (arrows, Fig. 5j, k). Furthermore, transverse sections from 1100 and 1400 d° revealed that C-4-S was arranged in the NS in concentric lamellae (Fig. 5i, l). The results indicate that C-4-S was arranged as a gradient, with strongest staining towards lumen and confined to more well-outlined structures, whereas DS showed a more widespread distribution. No staining for C-4-S was seen in the lumen of the notochord at any stages of development and in contrast to DS only weak staining could be detected in the chondrogenic zones.

### Chondroitin 6-sulphate (C-6-S) and unsulphated chondroitin (C-0-S)

**Fig. 6:**
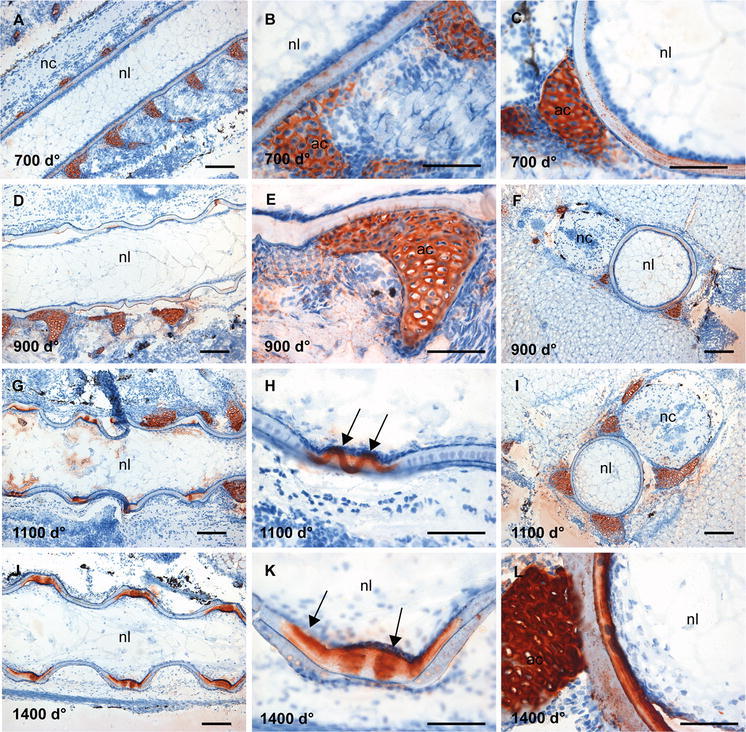
Distribution of C-6-S in the salmon spine. **a** Longitudinal sections at 700 d° shows a cartilaginous staining, and only weak label in the notochord sheath, enlarged in **b** with a granular layer close to the lumen. **c** Transverse section showing uneven staining of the notochordal sheath. **d** Longitudinal section at 900 d°, showing the same staining pattern as 700 d°, enlarged in **e**. **f** In transverse sections, staining was difficult to detect. **g** Longitudinal sections at 1100 d°, increased staining of the notochord sheath in the IVRs was seen, enlarged in **h** where a thread-like pattern of staining is evident, indicated by *arrows*. **i** Transverse section showing the staining of the arcualia. **j** Longitudinal section at 1400 d°, the staining remained strong and limited to IVRs, with strongest staining in a layer of the sheath parallel to the chordaepithelium, enlarged in **k** (indicated by *arrows*). **l** Transverse section showing strong staining towards the lumen and in cartilaginous tissue of the arches. *Scale bar* 100 µm; *nl* notochord lumen, *nc* neural cord, *ac* arch centra

The mAb 3B3 + cABC lyase, staining sulphate in the C-6-S position of the galactosamine, resulted in staining in the cartilaginous tissue of the arches at all developmental stages studied (Fig. 6) in mineralizing as well as unmineralized areas (Figs. 2a, d and 6a, d).

In contrast to C-4-S/DS, only weak label was detected in the sheath of the notochord at 700 d°, apparent as a granular layer localized above the chordaepithelium (Fig. 6a–c). At 900 d° (Fig. 6d–f), the stain was found mainly in the cartilages. During the segmentation process from 900 to 1100 d°, an increased labelling of the notochord was seen, restricted to the intervertebral ligaments (Fig. 6g–h), and similar circular structures as seen by use of mAb 2B6 after chondroitinase acII treatment appeared (arrows, Fig. 6h). At 1400 d°, the staining of C-6-S remained strongest in a layer of the sheath parallel to the chordaepithelium, limited to IVR, almost disappearing in the outer parts of the sheath (left arrow, Fig. 6k). In addition, two parallel circular structures, similar to those detected by 2B6 abc/ac in the longitudinal sections at 1100 and 1400 d° (Figs. 4k, 5h, k) appeared (right arrow, Fig. 6k).

**Fig. 7:**
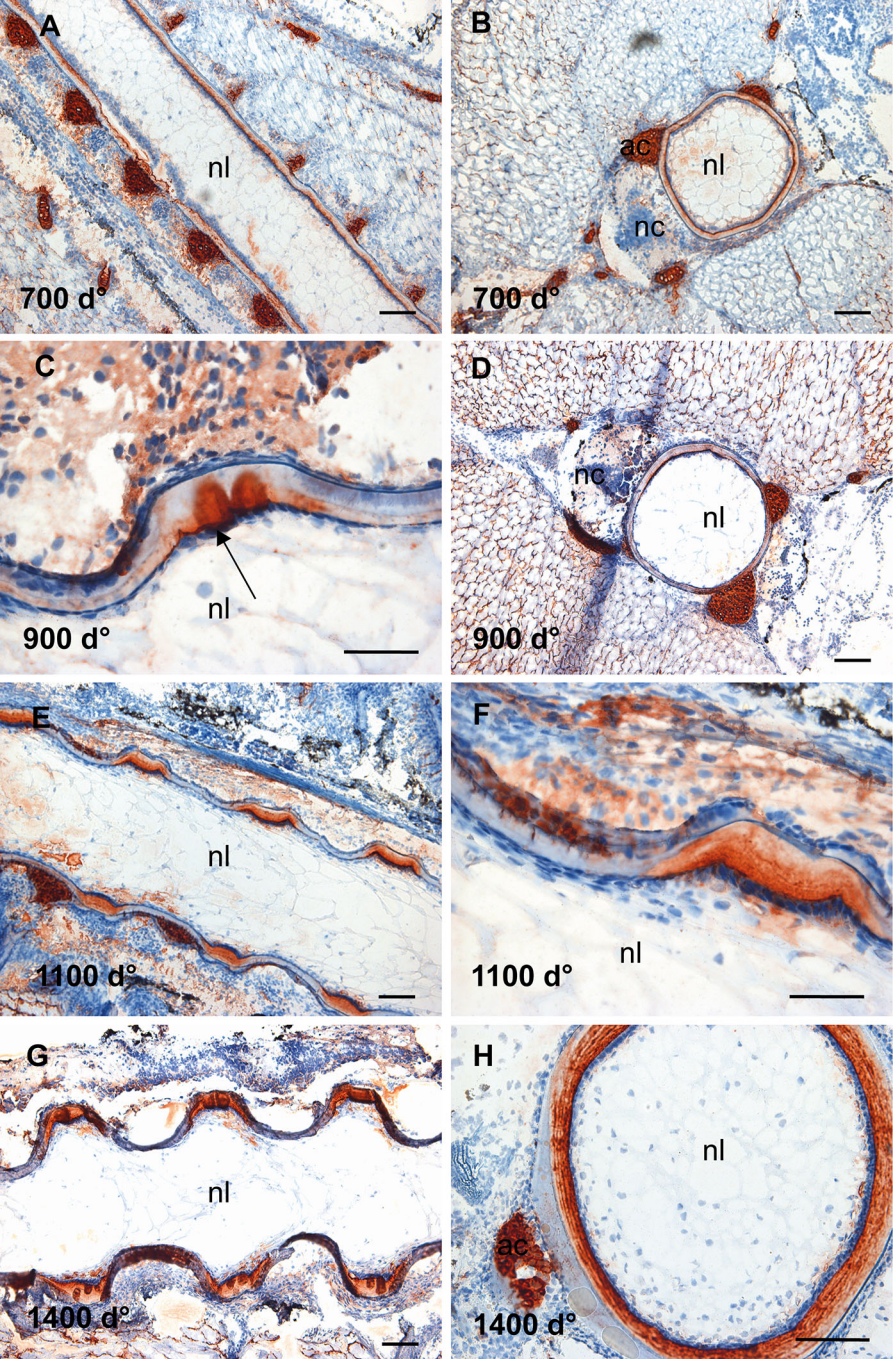
Distribution of unsulphated GAGs in the salmon spine. **a** Longitudinal section at 700 d° showing a widespread staining of the notochordal sheath, arcualia and in surrounding tissue. **b** Transverse section showing that the notochordal sheath stained stronger towards the lumen. Staining of arcualia is also clearly visible. **c** Longitudinal section at 900 d° showing distinct globule-like structures in the IVRs (*arrow*) and staining besides these globules in IVR closest towards lumen. **d** Transverse section at 900 d showing only weak staining. **e** Longitudinal section at 1100 d°, IVRs was stained and the transverse sections in **f** showed that the staining was strongest towards the lumen. **g** This was also evident of longitudinal section at 1400 d° and the transverse section in **h** show the distinct lamellar ring pattern at this stage. *Scale bar* 100 µm; *nl* notochord lumen, *nc* neural cord, *ac* arch centra

The mAbs 1B5 + cABC lyase, recognizing unsulphated GAGs, were expressed in the same areas as C-6-S and C-4-S/DS, indicating some co-distribution of sulphated and unsulphated GAGs in the arcualia and notochordal sheath (Fig. 7). But 1B5 + cABC lyase resulted in a stronger and more widespread staining of the notochordal sheath and surrounding tissue in sections from salmon collected at 700 d° compared to the mAbs against C-6-S and DS (Fig. 5). In addition, similar structures in the IVR as were outlined by the mAbs against the sulphated epitopes, C-4-S and C-6-S, appeared as early in development as 900 d° (arrow, Fig. 7). Also C-0-S was arranged in concentric lamellae in the transverse sections (Fig. 7h) in a similar way as C-6-S and C-4-S.

### Keratan sulphate (KS)

**Fig. 8:**
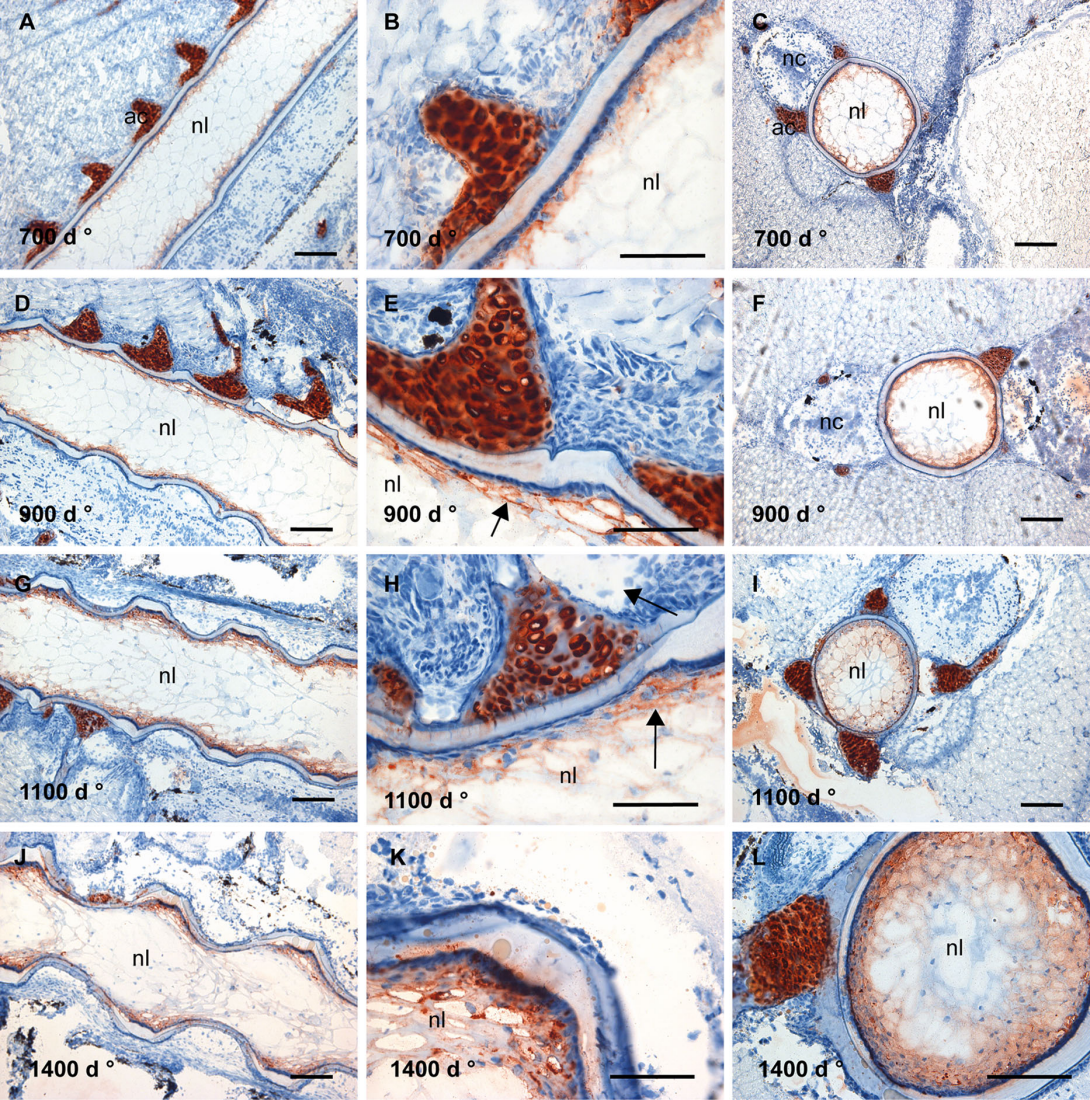
Distribution of KS in the salmon spine. **a** Longitudinal section at 700 d°, KS was detected in the periphery of the lumen of the notochord. In addition, strong staining was seen in the cartilaginous tissue of the arches. The enlarged **b** shows strong staining beneath the layer of chordaepithelium. **c** Transverse sections clearly show this pattern. **d** Longitudinal section at 900 d°, with chordoblasts staining, enlarged in **e** (indicated by *arrow*) and cartilaginous staining persisting. **f** Transverse sections clearly show this pattern. **g** Longitudinal section showing a highly evident increase in staining intensity, enlarged in **h** and indicated by *arrow*. **i** Transverse sections show highly increased staining at this stage in the lumen of notochord. **j** Longitudinal section at 1400 d°, showing that the intense staining in the lumen of notochord persisted, enlarged in **k**, and clearly visible in transverse section **l**. *Scale bar* 100 µm; *nl* notochord lumen, *nc* neural cord, *ac* arch centra

In contrast to CS and DS epitopes, KS labelling (using mAb 5D4) was detected primarily in the lumen of the notochord, beneath the layer of chordaepithelium (Fig. 8) as early in development as 700 d° (Fig. 8a–c). In addition, strong staining was seen in the cartilaginous tissue of the arcualia at all developmental stages. Both chordoblasts and chordocytes showed strong and consistent staining as seen in sections from fish of age 900 d° (arrow, Fig. 8e, h). An increase in staining intensity was seen during development, most evident beneath the chordaepithelium in the IVR areas (Fig. 8g–l). Only traces of staining could be seen in the NS (Fig. 8). This staining pattern indicates that another class of proteoglycans carries the KS epitopes.

### Distribution of GAGs in normal and malformed vertebrae from 15 g salmon

**Fig. 9:**
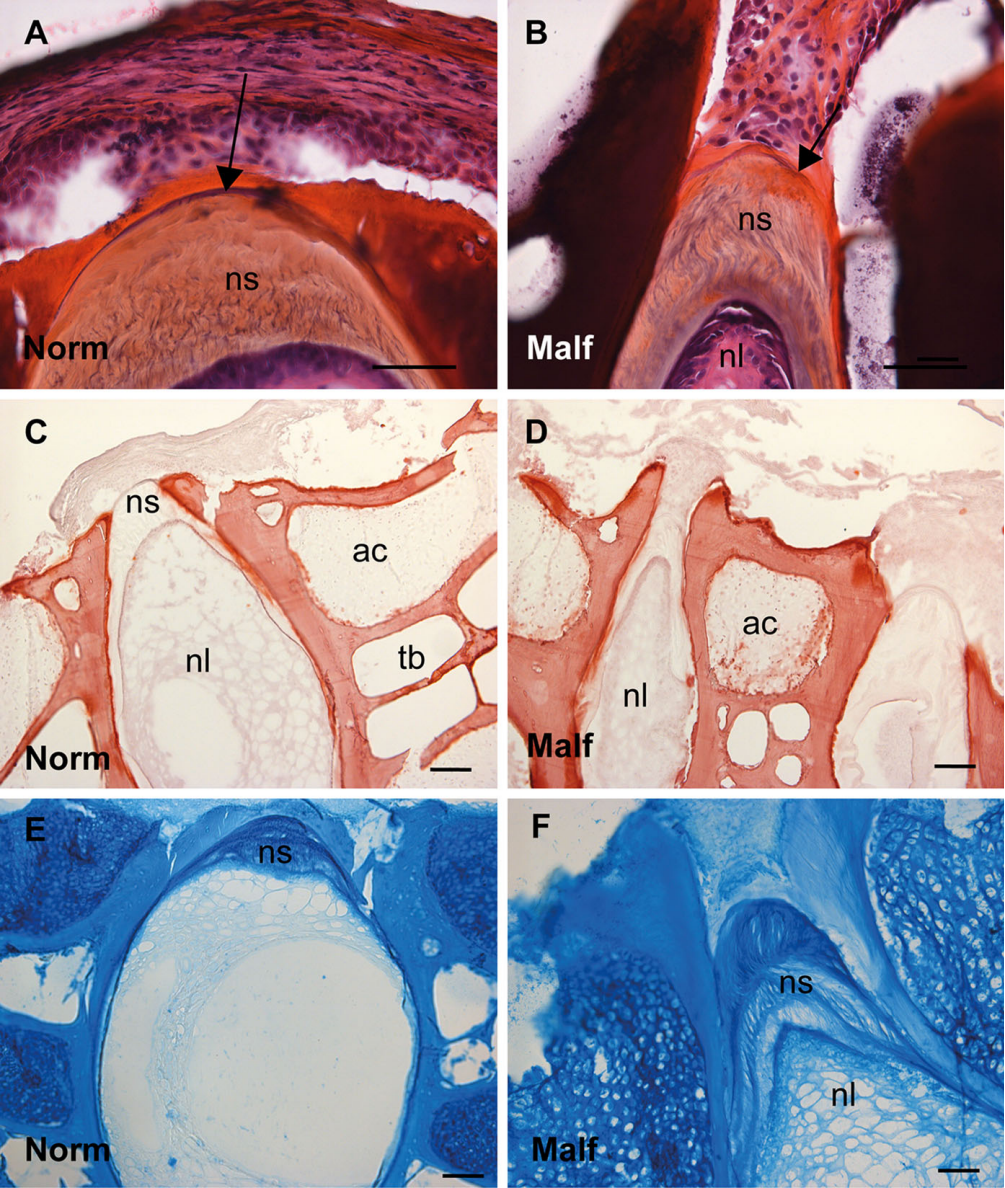
Histology of longitudinal sections of **a** normal and **b** malformed vertebrae from 15 g salmon. HE-added saffron staining clearly showing a weaker elastic lamina surrounding malformed vertebrae, as indicated by *arrows*, and a more elongated appearance containing wavy lamellar structures appearing more stretched in malformed notochordal sheath. Alizarin red S visualized thicker compact bone, flattened endplates and reduced IVRs, between **c** normal and **d** malformed. Alcian blue visualized a more homogenous GAG distribution in the notochordal sheath of **e** normal compared to **f** malformed. Different layers with weaker staining towards the lumen of the notochord and heavier stained chordoblasts can be observed in the malformed. *Scale bar* 100 µm; *nl* notochord lumen, *nc* neural cord, *ac* arch centra, *ns* notochordal sheath, *tb* trabeculae bone, *Norm* normal, *Malf* malformed

**Fig. 10:**
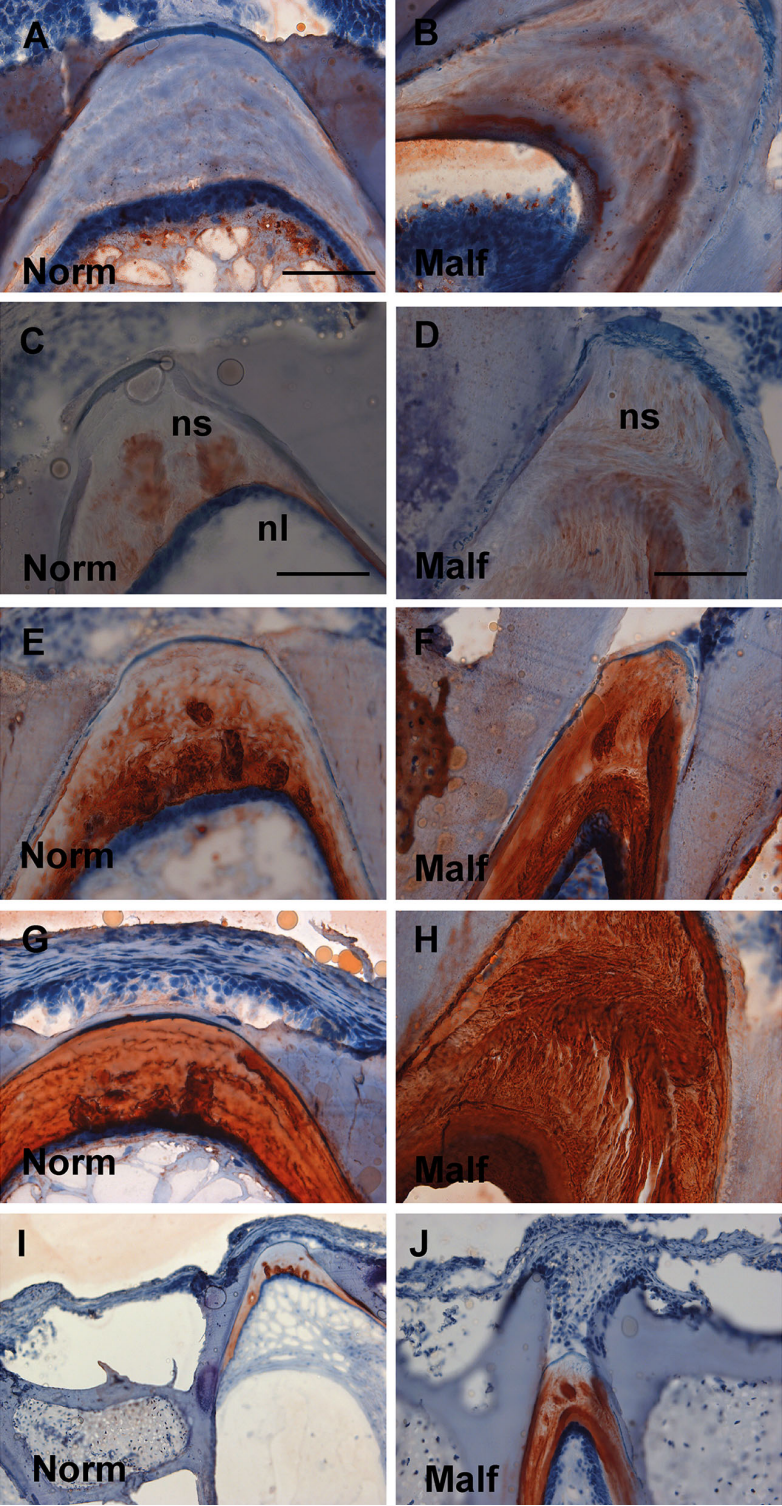
Immunostaining of different GAG types in longitudinal sections of normal and malformed vertebrae from 15 g salmon. *Left panel* shows normal vertebrae versus *right panel* shows malformed vertebrae. **a**, **b** KS was found mainly in the lumen of the notochord in normal and malformed, but also appeared strongly in the notochordal sheath in malformed. **c**, **d** C-6-S had distinct band with globular structures in the notochordal sheath of normal vertebrae, but these were lost in malformed vertebrae. **e**, **f** C-0-S was found closer to the lumen in the notochordal sheath of normal vertebrae in contrast to a more widespread distribution in malformed where this gradient was disturbed. **g**, **h** C-4-S/DS showed lamellar structures in the cranio-caudal direction of the notochordal sheath of normal vertebrae, but in malformed, these structures were ruptured and stretched more in the dorsoventral direction. **i**, **j** C-4-S showed weak staining in the notochordal sheath of normal vertebrae, but stained more intensively and in a spotted pattern in malformed vertebrae. *Scale bar* 100 µm; *nl* notochord lumen, *ns* notochordal sheath, *Norm* normal, *Malf* malformed

HE-added saffron staining of normal and malformed spinal columns showed a weaker external elastic lamina surrounding malformed vertebrae, as indicated by arrows in Fig. 9a, b. The notochordal sheath of malformed vertebrae also had a more elongated appearance containing vertical wavy lamellar structures (arrows, Fig. 9a, b). Alizarin red S visualized changes like thicker compact bone, flattened endplates and reduced IVRs, between normal and malformed vertebrae (Fig. 9c, d), as previous described in the literature (Ytteborg et al. 2010a). Alcian blue visualized a more homogenous GAG distribution in the notochordal sheath of normal vertebrae compared to malformed vertebrae. In the latter, Alcian blue stained the notochordal sheath in different layers, with weaker and intermittent staining in between mainly in the part of the sheath towards the lumen (Fig. 9e, f). Chordoblasts and chordocytes also stained heavier in malformed vertebrae (Fig. 9f), indicating more GAGs in the notochord of these individuals. The distribution of the GAG types and their organization patterns differed markedly between normal and malformed vertebral columns (Fig. 10). The major difference was seen in the distribution of KS epitopes. KS expression was present mainly in the lumen of the notochord of normal vertebral column (Fig. 10a), but also appeared strongly in the vertebral sheath of malformed samples (Fig. 10b). In addition, a more widespread distribution with changes in the organization pattern of CS epitopes in the intervertebral region was observed in the malformed vertebral column (Fig. 10d, f, h, j) compared to normal (Fig. 10c, e, g, i). The entire notochordal sheath in the malformed vertebral column showed intense staining and the staining pattern changed from parallel arranged lamellae to heavily stained, and more wavy irregular structures appeared. C-6-S had distinct band in the notochordal sheath of normal vertebrae; these were lost in malformed vertebrae (Fig. 10d).

### Western blotting

**Fig. 11:**
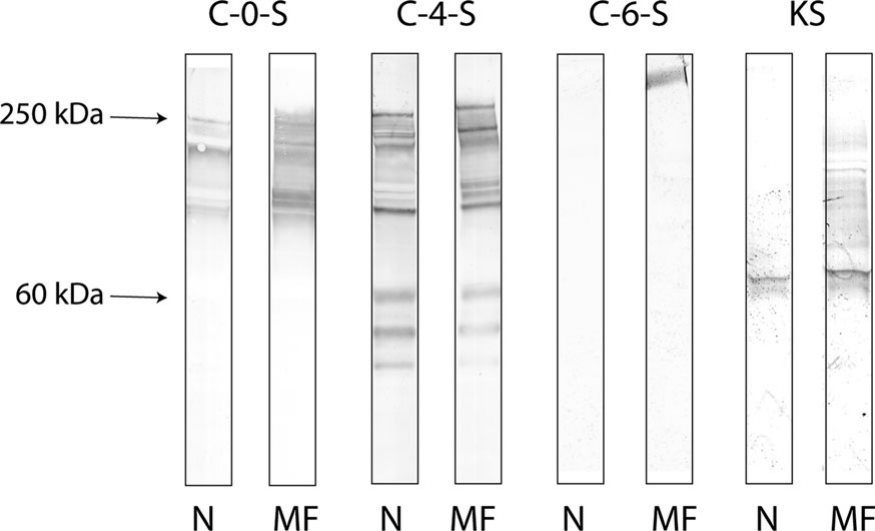
Western blotting of proteins isolated from normal and malformed vertebrae of 15 g salmon showing that C-0-S was primarily detected in molecules of higher sizes, whereas C-4-S showed additional bands in the region <60 kDa. Also the main band, appearing by mAb 5D4 against KS, exhibited a molecular size just below the 60-kDa marker. Some broad, weak stain was furthermore seen in the region for higher molecular sizes against KS, most clear in samples from malformed vertebra. mAb against C-6-S was only found this epitope in samples obtained from malformed vertebrae, appearing as a band in the region above the 250-kDa molecular marker. *N* normal, *MF* malformed

Proteoglycans represent a large family of molecules of different sizes which may carry same GAG chains. The results showed that C-0-S was primarily detected in molecules of higher sizes, whereas C-4-S showed additional bands in the region <60 kDa (Fig. 11). Also the main band, appearing by mAb 5D4 against KS, exhibited a molecular size just below the 60-kDa marker in accordance with the sizes for the small proteoglycans as decorin, biglycan, lumican and fibromodulin. Some broad, weak stain was furthermore seen in the region for higher molecular sizes, most clear in samples from malformed vertebrae. Regarding the mAb against C-6-S, we only found this epitope in samples obtained from malformed vertebrae, appearing as a band in the region above the 250-kDa molecular marker. The result suggests that this epitope is associated with PG with high molecular size, which changed into lower molecular size able to enter the gel during the pathological development.

### Q-PCR analyses

**Fig. 12:**
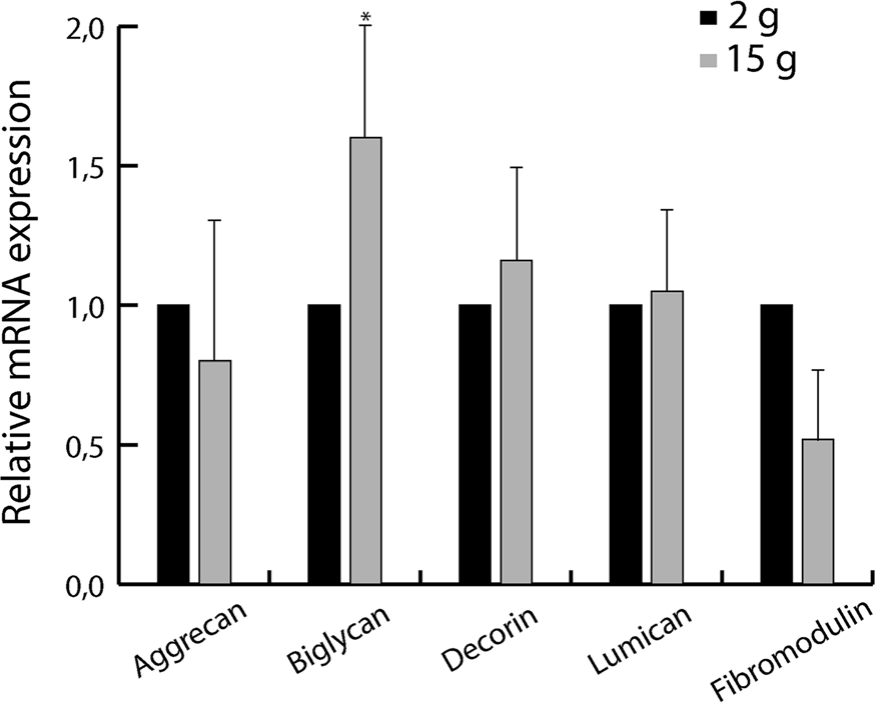
Transcription of aggrecan, biglycan, decorin, fibromodulin and lumican in the spinal column of 2 g (*n* = 12) and 15 g (*n* = 12) salmon. Biglycan transcription was the only gene that was significantly up-regulated. Levels of transcripts in triplicates are relative to elongation factor 1α (Ef1α), and data are presented as fold difference between 2 and 15 g fish. *Asterisk* indicates significant difference at *p* < 0.05 and *black bars* standard deviation

The results of the qPCR analysis of samples from non-deformed vertebrae collected from fish of 2 and 15 g are shown in Fig. 12. Transcription of the proteoglycans: aggrecan, biglycan, decorin, fibromodulin and lumican were found at both developmental stages. Comparison of the mRNA expression of the proteoglycans present in 2 and 15 g fish showed that biglycan transcription was the only one of the studied PGs which was significantly up-regulated during this period of growth. Decorin was also up-regulated, whereas aggrecan and fibromodulin transcription were down-regulated. None of these changes were significant. Lumican transcription was similar in samples from 2 and 15 g fish.

## Discussion

The present study describes the distribution of sulphated GAGs in the vertebral column of Atlantic salmon during development from 700 to 1400 d°, and in 15 g fish. During this period, the notochord evolves from a fire horse-like structure to a fully developed, segmented and mineralized vertebral column. This includes separation of the vertebrae, formation and insertion of ligaments and elaboration of synovial lining to occur simultaneously with continuing growth and mineralization. For all these processes to occur, many signals are required and must be highly coordinated and expressed in distinct patterns for proper differentiation and organization of the tissues involved. Disruption in any of these highly regulated processes may result in pathologic development, such as vertebral malformations.

Staining with Alcian blue demonstrated the presence of GAGs in the regions of growth and differentiation, in mineralized and unmineralized areas of the notochord, the arch anlagen and surrounding tissues at all developmental stages studied, suggesting a role for sulphated GAGs in vertebral growth and development in Atlantic salmon. The presence of GAGs as judged by the staining intensity of Alcian blue highly correlated with the Alizarin red S staining of calcified tissue, showing the presence of sulphated GAGs in all areas prior to mineralization, making a template for endochondral as well as intramembranous ossification. Intensive staining was also found in regions and tissues that do not mineralize, indicating different roles for the GAGs in growth and development of the vertebral column in salmon. Since Alcian blue with MgCl_2_ stains all highly anionic GAGs, structural differences in the various subtypes were approached by immunohistochemistry after enzymatic digestion. Our study showed that in the mineralizing areas of the arches, DS, C-0-S, C-6-S and KS were strongly expressed at 700 d° and the staining persisted until adulthood. In contrast, in the notochord sheath, the different GAG epitopes showed changing temporal and spatial distribution during development. The most striking changes in the composition of the GAGs occurred in the IVR, suggesting a role for GAGs also in the patterning of the vertebral column.

At 700 d°, the IVR was part of a uniform-stained sheath of even thickness, consisting primarily of DS. The staining pattern for DS appeared granulated, but was later arranged in IVR as longitudinal layers. Grotmol (Grotmol et al. 2006) has previously described the presence of parallel Collagen 2 fibrils in the notochordal sheath, forming helices around the longitudinal axis of notochord during early life. Thus, the granular staining pattern may be due to DS chains associated with SLRPs, surrounding the collagen fibrils. At later, developmental stages DS were arranged also in parallel layers in the IVRs running from the anterior to the posterior endplates, indicating presence of GAGs in fibres connecting the vertebral bodies.

At 900 d°, mineralization of the chordacentra was completed and the composition of the GAGs changed. We observed a folding or bulging of the sheath in the regions were IVR developed and circular structures containing C-4-S, C-6-S and C-0-S epitopes appeared. Using mAb against C-6-S, a worm-like structure appeared in the sections collected at 1100 d°. The structure most likely represents the same circular structures seen in IVR in sections from previous developmental stages, and the observed differences may be a result of minor variations in the para-sagittal plane during sectioning. The functional role of the structures is unknown. However, they were discussed in Ytteborg et al. (2010a), and a filtering role for these structures, by removing waste products and providing nutrition to and from the notochord, was suggested. They may represent vessels and channels for transportation, in a similar way as the angiogenesis (vasculogenesis) described in the distal femoral chondroepiphysis of rabbit during development and ossification (Doschak et al. 2003) and in human lumbar intervertebral discs during development from foetal to infantile age (Nerlich et al. 2007).

The appearance of globes and bulges in the IVR in this experiment occurred simultaneously with completed circumferential mineralization of the chordacentra, thus, at a time where growth, size and structure might demand for a more organized and effective transportation system. During malformation, the epitopes were arranged in dorsoventral waves, ruptures or fissures in between the waves, indicating a higher level of degradation of matrix components associated with the collagenous network in the notochordal sheath. The globular structures containing C-6-S epitopes in the healthy IVR could not be detected in the malformed IVR, suggesting a change in these structures during the pathogenesis, which may possibly alter the nutritional access of the notochord during deformities.

An increase in the staining for C-6-S in the IVR seen with development has also been reported in murine vertebrae (Hayes et al. 2001). Staining for C-6-S was found in the regions above the chordaepithelium and in the interface between the soft tissue of the IVR and the bony endplates. The former region has to restrict the pressure from the expanding vacuolated chordocytes (Grotmol et al. 2005). During the period examined, stiffening of the vertebral column occurs due to mineralization of the chordacentra, resulting in greater mechanical challenges on the IVRs, which also have to resist bending movements and compression during swimming. C-6-S is usually a constituent of hyalectans: forming large water binding aggregates and enabling the tissue to resist compression. Important information on the role of C-6-S in bone development has come from recent study on humans with spondyloepiphyseal dysplasia (Omani type) with a missense in the gene encoding for the enzyme C-6-S T-1 producing C-6-S. Severe progressive scoliosis preceded by diminished intervertebral discs and abnormal vertebral endplates, accompanied by severe arthritic changes with joint dislocations (Thiele et al. 2004). Thus, C-6-S most likely plays a similar role in vertebral development in teleosts as Atlantic salmon. Interestingly, we found increased staining intensity in the malformed IVRs. However, the structure of the sheath seemed to be different from what was found in normal notochordal sheath, indicating a compensatory role of increased protein production in these regions during compression.

The major GAG present in the notochord sheath and arches at 700 d° was 4-sulphated DS as only traces of staining from cACII generated epitopes were detectable. DS is a structural isomer of CS, in which some of the glucuronate residues are epimerized to iduronic acid (IdoUA). The epimerization has an important effect on the binding properties of GAGs, as IdoUA can take up more than one ring confirmation, thus increasing the flexibility of the GAG chain (Casu et al. 1988). DS PGs, such as decorin and biglycan, are reported to have a widespread distribution in mammalian tissue such as blood vessel walls, skin, tendon cartilage and undifferentiated mesenchymal tissue (Rosenberg et al. 1985) where they participate in extracellular matrix organization, neurite outgrowth, wound repair, cell adhesion, migration and proliferation, promoting growth factors (Trowbridge and Gallo 2002) (Taylor et al. 2005). In the present study, we demonstrated an up-regulation of biglycan mRNA during development from 2 to 15 g. Biglycan is reported to control signalling pathways regulating the osteogenic program (Berendsen et al. 2011), and in vitro studies have demonstrated a different influence of biglycan and decorin on mineralization (Mochida et al. 2003). A changed mRNA expression of decorin and biglycan has previous been demonstrated in salmon of 15 g size with vertebral deformities (Pedersen et al. 2013). Thus, the presence of four sulphated DS in the notochord during growth and development of salmon is most likely a prerequisite for proper development, but further studies are needed to explore the consequences of the differences in biglycan and decorin expression in vertebral development. In malformed vertebrae, we found that immunolocalization of CS/DS epitopes changed in the vertebral sheath from parallel arranged lamellae to heavily stained, and more wavy vertical irregular structures appeared.

In the present study, C-4-S epitopes created by cACII treatment showed a distinct expression pattern, emerging later during development and restricted to the IVR, indicating specific role for this GAG in this particular region of the notochord. It is reported that CS plays important modulatory role on proteinase activities (Georges et al. 2012). Activation of pro-MMP-2 by MMP-16 was significantly enhanced in the presence of excess C-4-S, whereas C-6-S was ineffective. CS-GAGs did also participate in regulating bone resorption through modulation of cathepsin K activity (Li et al. 2002). Moreover, at acidic pH, the collagenase activity of cathepsin K was enhanced in bone and cartilage by CS and KS, whereas DS and HS selectively inhibited its activity (Li et al. 2004). Hence, the distinct pattern of C-4-S in the IVRs may indicate an inhibitory role of C-4-S in bone formation in the developing notochord of growing Atlantic salmon, ensuring flexibility of the vertebral column by preventing bone and cartilage formation in these regions.

KS was hardly detected in the IVR at any stages of development, neither in the globular structures nor in the matrix between. However, KS was present in the lumen of the notochord at all developmental stages studied. The labelling for KS increased in this area during the life span from 700 to 1400 d°, located mainly in the vacuoles of an increasing number of chordocytes. In notochord, the chordoblasts produce the matrix of the sheath and continue to divide throughout life in accordance with sustained notochordal growth (Grotmol et al. 2006). They further maturate into chordocytes, containing large fluid filled vacuoles which functional role is to maintain internal hydrostatic pressure (Adams et al. 1990; Glickman et al. 2003; Nordvik et al. 2005). KS is known to be the GAG with the highest water binding capacity, and PGs carrying KS chains are considered to regulate water balance of the extracellular matrix. Thus, a role for KS contributing to the hydrostatic pressure of the notochord of Atlantic salmon by vacuole formation is plausible. KS may be a part of aggrecan, a hyalectan, known to form huge aggregates with hyaluronic acid. Aggrecan was identified in the chordocytes of Atlantic salmon notochord (Ytteborg et al. 2010b). Mammalian aggrecan carries a large number of GAG chains with C-6-S epitopes in addition to KS. In our study, C-6-S did not co-localize with KS in the chordocytes, suggesting the presence of other KS PGs. In a recent study, we immunolocalized lumican in the chordocytes of Atlantic salmon (Pedersen et al. 2013). Lumican is reported to carry KS also in teleosts such as Atlantic cod (Tingbo et al. 2012) and zebrafish (Souza et al. 2007).

In contrast to the healthy vertebral column, KS was expressed throughout the entire sheath from the lumen towards the external lamina in malformed vertebral columns, showing a similar wavy distribution pattern as C-4-S/DS and C-6-S. The co-distribution of KS and C-6-S epitopes indicated the presence of aggrecan in the notochord sheath and may reflect a reaction to resist increasing mechanical challenges due to malformation (Witten et al. 2006). Production of KS is also reported in the lack of O_2_ indicating disturbance in O_2_ supply. Moreover, SDS and western blot showed more breakdowns of the molecules carrying these epitopes during the pathological process. In contrast to healthy vertebrae, C-6-S epitopes from malformed vertebrae were able to penetrate the gel. Increased staining pattern of high molecular weight compounds were observed on the blots also for C-0-S and KS epitopes. In a previous study, an up-regulation at mRNA level of metalloproteinases (MMP-9), an enzyme able to degrade PGs (Genovese et al. 2013), was demonstrated in malformed vertebrae from Atlantic salmon (Ytteborg et al. 2010a). In the mammalian IVD, a breakdown of aggregating PG components like aggrecan is related to loss of hydrostatic pressure, tissue dehydration and disc degeneration (Brzoska and Moniuszko-Jakoniuk 2001; Kauppila 1995; Urban and Mcmullin 1985; Yasuma et al. 1993).

## Conclusion

The present study has outlined the temporal and spatial distribution of different sulphated GAG epitopes, 4-sulphated CS/DS, KS, C-0-S and C-6-S in the Atlantic salmon spinal cord, both in a developmental perspective and in the light of vertebral malformation. The expression of these components was confined to distinct regions of the notochord during the period when the vertebral bodies form and mineralize. The results showed a persistent spatial distribution pattern of the GAGs from 1100 d° to 15 g, indicating similar functions for the different GAGs both in the juvenile and in the mature spine. This pattern was disrupted in vertebral malformations, exhibiting gross alterations in the composition of the ECM accompanied with an increase in matrix degradation during the pathogenesis. Increased lamellar disorganization and fissures in the IVR were features of vertebral malformation. More GAGs was produced in the matrix of notochord during the initial stages of pathogenesis judged by the stronger staining with Alcian blue. We suggest that GAGs are important for normal sheath integrity and that altered matrix composition is of importance for the pathogenesis.
